# Retrospective cohort study on long-term anticoagulation therapy for bleeding complications among atrial fibrillation patients

**DOI:** 10.6026/973206300213417

**Published:** 2025-09-30

**Authors:** Megha Joy, Bethamcherla Bala Chandrudu, Mohamed Thoufeek Hussain Amjat Ibrahim, Richard Fleming, Harish Sidharth Veerachamy

**Affiliations:** 1 Department of Internal Medicine, Queen Elizabeth Queen Mother Hospital, Margate, United Kingdom; 2 Department of Medical Physiology, Visvabharathi Medical College and General Hospital, Penchikalapadu, Andhra Pradesh, India; 3 Department of Vascular Surgery, Apollo main hospital, Karpaga Vinayaga Institute of medical science and research center, Tamil Nadu, India; 4 Department of General Medicine, Snehadaan Hospital, Bengaluru, Karnataka; 5 Department of General Medicine, Tirunelveli Medical College, Tamil Nadu, India

**Keywords:** Warfarin, DOACs, anticoagulation, bleeding risk, atrial fibrillation, INR fluctuation, long-term treatment

## Abstract

The frequencies and contributing factors to bleeding complications among atrial fibrillation patients on a regimen of long-term oral anticoagulants is of interest. The medical records for 142 patients treated with either warfarin or direct oral anticoagulants (DOACs) were assessed over a period of three years. Major and minor bleeding episodes were associated with instability of the international normalized ratio (INR), the type of anticoagulant medication, and individual patient characteristics. The bleeding complications were observed to occur more frequently with patients presenting with unstable INR values and those treated with warfarin in contrast to DOACs patients. Thus, the importance of a tailored anticoagulation regimen to address bleeding complications in atrial fibrillation is shown.

## Background:

Due to their very wide range of indications, anticoagulants are one of the most commonly used drug groups. Although these drugs are characterized by different mechanisms of action, the most common complication of their use is still bleeding episodes, the frequency of which depends largely on the clinical condition of the patient using such therapy [1]. One prevalent cardiac arrhythmia linked to an increased risk of thromboembolic events, including ischemic stroke, is atrial fibrillation (AF) [2]. One of the mainstays of managing atrial fibrillation (AF) is long-term oral anticoagulation therapy, which uses direct oral anticoagulants (DOACs) or vitamin K antagonists like warfarin [3]. However, the higher risk of bleeding complications which can vary from modest mucosal bleeding to potentially fatal hemorrhages-must be weighed against the clinical benefit of preventing stroke [4]. DOACs provide more predictable pharmacokinetics, but warfarin's limited therapeutic window and multiple drug-food interactions necessitate frequent INR monitoring [5]. Real-world evidence on bleeding risk over long periods of time is still inconsistent despite their extensive use, especially in varied patient populations [6]. Therefore, it is of interest to determine clinical factors linked to these unfavourable outcomes and to retrospectively assess the prevalence and trends of bleeding problems in AF patients undergoing long-term anticoagulation treatment.

## Materials and Methods:

Using tertiary cardiac center medical records, this retrospective cohort analysis examined cases from January 2020 to December 2023. The study comprised 142 adult patients with non-valvular atrial fibrillation who had been receiving oral anticoagulation medication continuously for more than a year. Based on the anticoagulant they were taking, patients were split into two groups: 78 were on Warfarin, and 64 were on DOACs, which include apixaban, rivaroxaban, and dabigatran. Exclusion criteria included patients with prosthetic heart valves, concurrent dual antiplatelet therapy, advanced liver disease, or known bleeding disorders. Data collected included demographic details, comorbidities, CHA_2_ DS_2_-VASc and HAS-BLED scores, type and duration of anticoagulant therapy, INR values (for warfarin), and incidence of both major and minor bleeding events. Outcomes were assessed using descriptive and inferential statistical methods, with logistic regression used to identify predictors of bleeding complications.

## Results:

Bleeding complications were more frequent in patients on warfarin compared to those on DOACs, with major bleeding events significantly associated with higher HAS-BLED scores and unstable INR levels. Minor bleeding was common across both groups but less severe in DOAC users. Predictive analysis confirmed that elevated age, renal dysfunction, and history of bleeding were strong independent predictors. Table 1 (see PDF) shows the study population had similar demographic and clinical risk factors across both treatment groups. Table 2 (see PDF) shows the incidence of bleeding (both major and minor) was higher in the warfarin group. Table 3 (see PDF) shows among major bleeding events, gastrointestinal bleeding was the most common. Table 4 (see PDF) shows Epistaxis, gum bleeding, and easy bruising were the most frequent minor events. Table 5 (see PDF) shows Time in therapeutic range (TTR) for warfarin was suboptimal in many cases, contributing to bleeding risk. Table 6 (see PDF) shows Bleeding rates increased significantly in warfarin users with TTR <50%. Table 7 (see PDF) shows renal dysfunction and advanced age were independently associated with major bleeding risk. Table 8 (see PDF) shows the frequency of monitoring was greater in the warfarin group due to INR variability. Table 9 (see PDF) shows Patients on DOACs had better therapy adherence scores. Table 10 (see PDF) shows Hospital admissions due to bleeding were predominantly in the warfarin group.

## Discussion:

This retrospective study demonstrates that long-term anticoagulation in atrial fibrillation patients carries a significant risk of bleeding, particularly in those receiving warfarin [7]. Major bleeding events were notably higher in patients with elevated HAS-BLED scores, impaired renal function and poor INR control (TTR <50%) [8]. While warfarin remains widely used, its effectiveness is heavily dependent on regular monitoring and patient adherence [9, 10]. In contrast, DOACs showed a safer bleeding profile, higher adherence and fewer hospitalizations, making them a preferable choice in many real-world scenarios [11, 12]. The findings support growing evidence that DOACs offer a favorable balance between stroke prevention and bleeding risk, particularly in elderly and high-risk populations.

## Conclusion:

Long-term anticoagulation among AF patients requires individualized risk assessment to minimize bleeding complications. DOACs demonstrate superior safety and adherence compared to warfarin, especially in patients with unstable INR or high HAS-BLED scores. Optimizing anticoagulation strategies is crucial to enhancing therapeutic outcomes while reducing harm.
